# Ultrasound Characteristics of Twisted Pedunculated Subserosal Uterine Leiomyomas: A Case Report and a Systematic Review of the Literature

**DOI:** 10.1002/jcu.70292

**Published:** 2026-06-08

**Authors:** Maddalena Paoli, Pietro Pasquini, Alessandra Pennetta, Ghizzi Chiara, Maria Cristina Scifo, Evaldo Giacomucci

**Affiliations:** ^1^ Department of Medical and Surgical Sciences (DIMEC) University of Bologna Bologna Italy; ^2^ Division of Oncologic Gynecology IRCCS Azienda Ospedaliero‐Universitaria di Bologna Bologna Italy; ^3^ Maternal Newborn and Child Department AUSL Bologna Bologna Italy; ^4^ Infertility Gynecology Program, Ospedale Maggiore AUSL Bologna Bologna Italy

**Keywords:** pedunculated leiomyomas, torsion, transvaginal ultrasound, ultrasonography

## Abstract

Torsion of pedunculated subserosal uterine leiomyomas (TPSUL) is a rare but potentially severe condition that may require emergency surgery. This article aims to summarize the existing literature by presenting a systematic review and an illustrative case report. A total of 38 articles describing 42 cases of TPSUL were analyzed to identify recurring ultrasound features that may aid in preoperative diagnosis. The most frequently reported findings included a mean maximum diameter of approximately 10 cm, increased size compared to prior scans (100%), heterogeneous echotexture (81%), regular borders (90%), and posterior or trans‐lesional acoustic shadows (94%). Additional common features were the presence of free pelvic fluid (57%), cystic areas with irregular margins and hypo‐anechoic content (55%), and absent (68%) or reduced (32%) vascularization of the lesion. A vascular pedicle was identified in 28% of cases, with signs of torsion in 12.5% and interruption of vascular flow at the pedicle base in 25%. The ultrasound features observed in our case, involving a 29‐year‐old pregnant patient at 21 weeks' gestation, were consistent with those described in the literature. These findings support the role of ultrasound as a useful tool for the preoperative identification of TPSUL and lay the foundation for future diagnostic standardization.

## Graphical abstract

Ultrasound may aid in the preoperative diagnosis of torsed pedunculated subserosal uterine leiomyomas (TPSUL), a rare but urgent condition. This study identifies consistent sonographic features across 42 cases, supporting ultrasound as a valuable diagnostic tool and highlighting the need for standardization in TPSUL imaging.

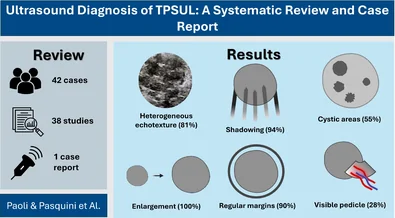

## 1 Introduction

Uterine leiomyomas, commonly known as myomas or fibroids, are the most frequent benign tumors of the female genital tract, being found in 40%– 89% of the female population of reproductive age (Savelli and Cristina Scifo 2015; Marsh et al. 2024). They develop within the uterine wall and originate from the clonal expansion of a myometrial cell (Bulun 2013). Their growth depends on hormonal stimulation (estrogens and progestins), which makes them rare before menarche and causes them to regress after menopause (Marsh and Bulun 2006; Andersen 1996).

Among the most widely used classifications, the FIGO system revised in 2018 is the leading one, categorizing leiomyomas into Types 0–8 based on their relationship with the endometrium, myometrium, and serosal surface, and therefore on their anatomical position within the uterus. Of particular interest for this study are Type 7 myomas, which correspond to subserosal pedunculated leiomyomas (Munro et al. 2018).

Subserosal pedunculated myomas are generally asymptomatic, although they may present symptoms due to compression of surrounding organs (rectum, bladder) or if they undergo degeneration due to inadequate blood supply, as in cases of rapid growth or torsion on their pedicle (Savelli and Cristina Scifo 2015; Gupta and Manyonda 2009).

Torsion of a subserosal pedunculated fibroid is one of the acute complications related to leiomyomas, with symptoms varying in severity depending on the degree of rotation and the speed at which torsion develops, often manifesting as acute pelvic pain (Gupta and Manyonda 2009; Roy et al. 2004). This is a rare occurrence: in a retrospective study conducted in Taiwan between 2001 and 2015, the percentage of cases of torsion among patients with subserosal uterine leiomyomas who underwent surgery was estimated to be less than 0.25% (Lai et al. 2018). Given this, healthcare providers generally have limited personal experience, making theoretical knowledge necessary to formulate a clinical suspicion.

From a histological perspective, leiomyomas can present various histopathological patterns of degeneration, including hyaline, cystic, myxoid, or hemorrhagic degeneration. The acute and complete torsion of a subserosal pedunculated leiomyoma initially leads to circulatory stasis, venous congestion, and edema. Consequently, there is compression of the arterial network, creating an obstacle to blood supply to the fibroid. The result is infarction of the leiomyoma, accompanied by hemorrhagic degeneration and tissue necrosis. If prompt intervention is not taken, superinfection and peritonitis may occur (Gupta and Manyonda 2009; Roy et al. 2004; Testa et al. 2016; Franco et al. 2023; Le et al. 2019). More rarely, rupture of the fibroid may also occur, leading to subsequent hemorrhagic shock (Chen et al. 2019). Acute torsion of a pedunculated leiomyoma thus represents a surgical emergency that requires an accurate diagnosis and timely intervention. To date, there are no studies in the literature that address the ultrasound (US) diagnosis of twisted pedunculated subserosal uterine leiomyomas (TPSUL) in a standardized manner. The gold standard for diagnosis is represented by the intraoperative findings, due to the limited diagnostic capability achieved so far with imaging techniques (Lai et al. 2018; Marcotte‐Bloch et al. 2007). Myomectomy is generally curative, but the real challenge is the preoperative diagnosis (Gupta and Manyonda 2009).

US combined with Doppler technique is an effective method for detecting, mapping and characterizing leiomyomas, providing both qualitative and quantitative analysis of vascularization (Savelli and Cristina Scifo 2015; Testa et al. 2016). This procedure is readily available, quick and preferable to CT and MRI, as it is significantly more cost‐effective, does not involve ionizing radiation and can be performed by an Obstetrics‐Gynecology Emergency physician. Transabdominal US is often preferred over transvaginal technique for visualizing subserosal pedunculated myomas, especially if they are large and extend into the abdominal cavity (Testa et al. 2016; Gassama et al. 2017; Foissac et al. 2011) or in the case of a gravid uterus. Typically, uncomplicated myomas appear as rounded or ellipsoid hypoechoic masses with a pseudocapsule that presents as a thin outer hyperechoic border (Savelli and Cristina Scifo 2015).

US easily identifies the presence of subserosal myomas, whereas the sonographic diagnosis of torsion can be highly challenging. For this reason, we focused our efforts on identifying direct and indirect US signs that could guide toward this diagnosis.

## 2 Case Report

We present the case of a 29‐year‐old primigravida at 21 weeks of gestation, with a TPSUL diagnosed via US. She presented to the AUSL Ospedale Maggiore di Bologna, Italy, in October 2023 for acute abdominal pain. The patient underwent a physical examination, laboratory investigations and transabdominal US. She was subsequently hospitalized for careful clinical and laboratory monitoring and was conservatively treated with electrolyte and analgesic therapy, with beneficial results. She was discharged 48 h after admission with home analgesic therapy. A follow‐up US examination was recommended but was not performed, as the pregnancy was subsequently monitored at another referral center. In February 2024, the patient underwent a cesarean section and concurrent myomectomy at the other referral center; the diagnostic suspicion of TPSUL was confirmed by intraoperative findings and histological analysis.

At the time of admission, a transabdominal US study was performed using the Esaote MyLabXPro80 US machine (Figures 1 and 2) at the Gynecological Emergency Acceptance Clinic of Ospedale Maggiore, Bologna. Subsequently, on the same day, the patient was re‐evaluated with a transabdominal US performed with the Voluson E8 US machine at the Gynecological Ultrasound Clinic of Ospedale Maggiore, Bologna (Figures 3 and 4).

**FIGURE 1:**
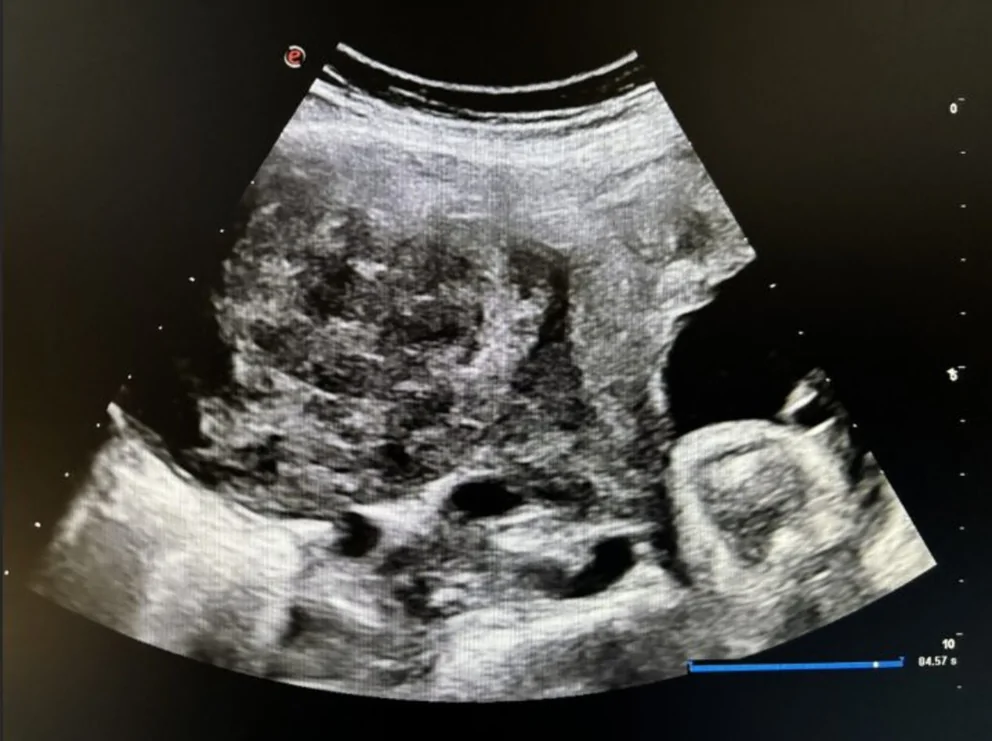
Image obtained with the Esaote MyLabXPro80 ultrasound machine. The mass showed heterogeneous echostructure and regular margins. Within the mass, cystic areas were observed, characterized by irregular borders and low‐level content.

**FIGURE 2:**
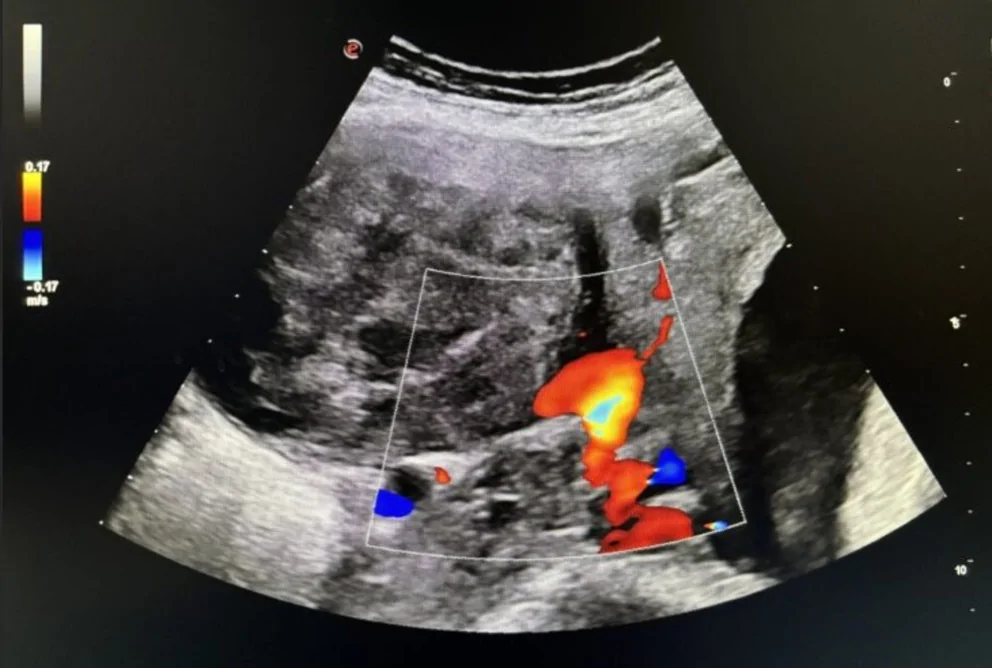
Image obtained with the Esaote MyLabXPro80 ultrasound machine. Upon application of Power doppler (PD), vascularization was absent within the fibroid (CS 1), while blood flow was clearly visible in the pedicle, which contained a vascular axis and appeared twisted upon itself.

**FIGURE 3:**
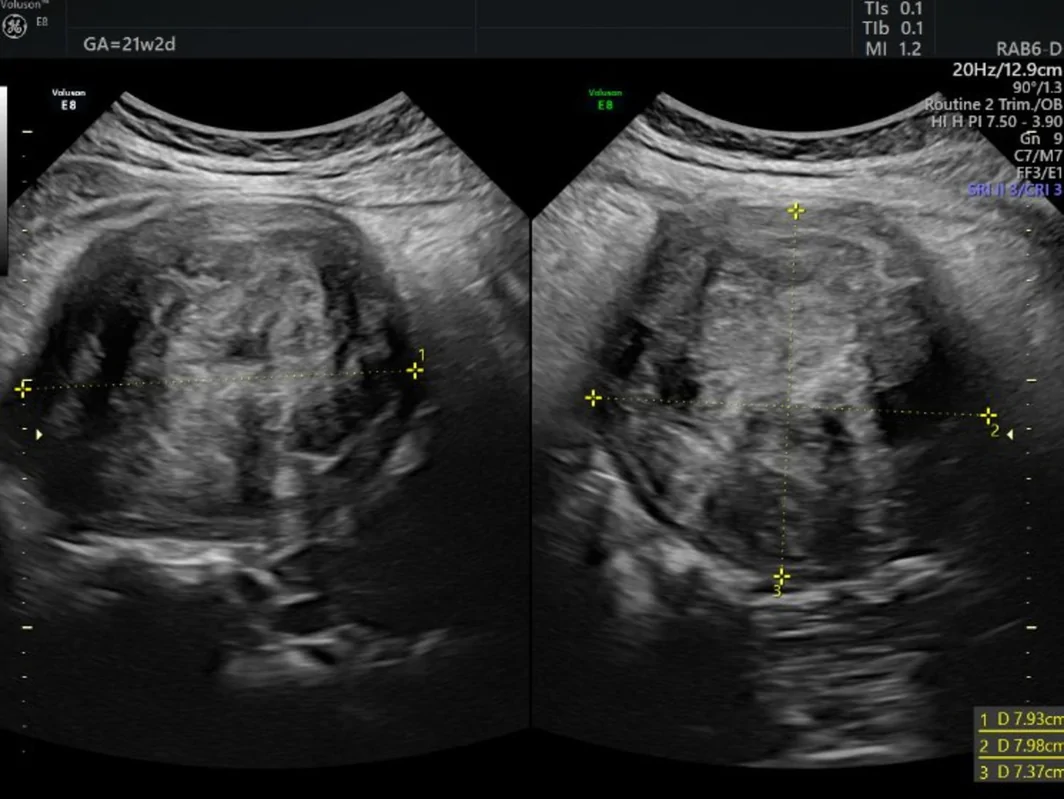
Image obtained with the Voluson E8 ultrasound machine. The mass measured 79 × 80 × 74 mm and showed thin posterior and through‐lesion acoustic shadows. As in the images obtained with the Esaote MyLabXPro80, the external margins were regular and the echostructure was heterogeneous, with irregularly contoured cystic areas of reduced echogenicity.

**FIGURE 4:**
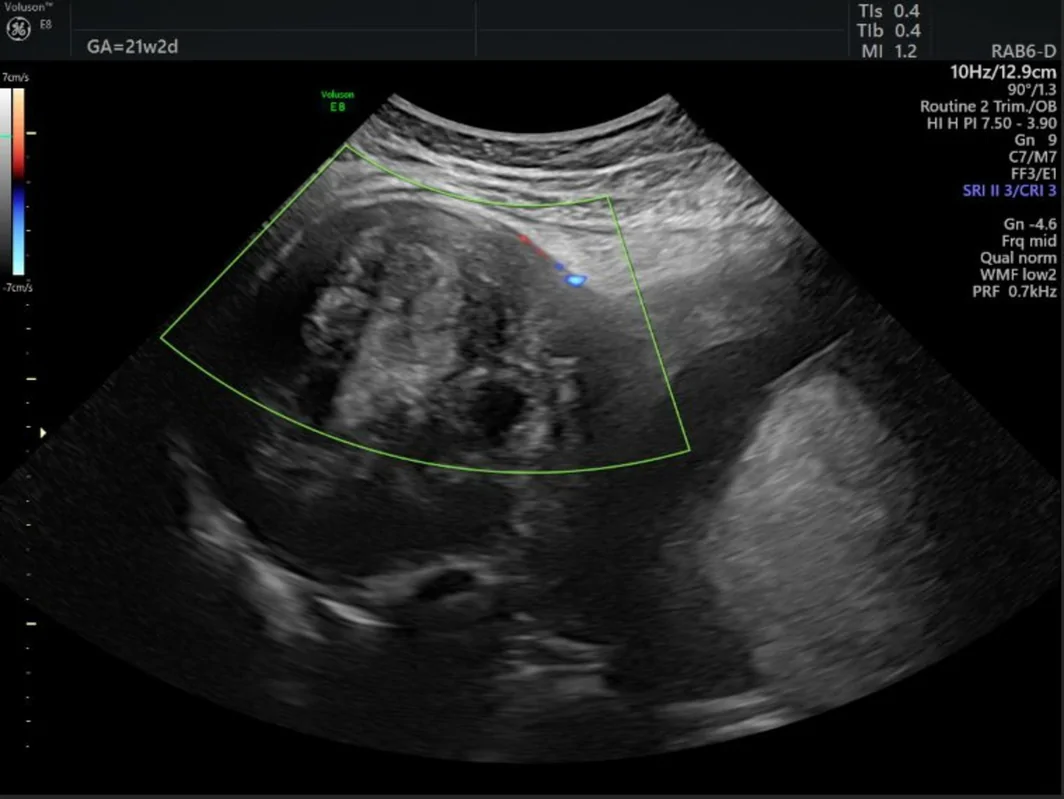
Image obtained with the Voluson E8 ultrasound machine. Upon application of Power Doppler (PD), vascularization was absent within the fibroid (CS 1).

The US study revealed a rounded hypoechoic solid mass on the right lateral wall of the uterus, hypomobile in relation to the uterus and painful upon targeted pressure with the probe, compatible, at first glance, with a pedunculated subserosal fibroid (Type 7 according to the FIGO classification) (Munro et al. 2018). The mass measured 79 × 80 × 74 mm and showed a heterogeneous echostructure with regular external borders. Cystic areas with irregular contours and low‐level content were visible within the mass. The Power Doppler examination revealed absence of vascularization within the fibroid, while blood flow was observed in the connecting pedicle, which contained a vascular axis and appeared twisted. Acoustic shadows, both posterior and through‐lesion, were also noted. A small amount of pelvic fluid was visible. The most illustrative images were selected and reported (Figures 1, 2, 3, 4).

## 3 Methods

Regarding the review of the literature, the searches were conducted on PubMed, Scopus, and Google Scholar. The databases were consulted from the beginning up to February 1, 2024. The keywords used for the search are listed in Table 1. Additionally, the references of all included studies were manually reviewed to identify further relevant documents.

**TABLE 1:** Literature search strategy.

Search strategy
1. “Fibroid*” OR “Myom*” OR “Leiomyom*” OR “Uterine neoplasm*”
2. “Torsion*” OR “Torsed” OR “Twist*”
3. “Ultrasound*” OR “Ultrasonograph*” OR “Sonograph*” OR “Sonogram” OR “Echotomography” OR “Ultrasonic”

*Note:* Search strings 1, 2, and 3 were combined using the Boolean term AND. Medical Subject Headings (MeSH) terms were also incorporated where applicable. Then the limits were applied.

The initial search resulted in a total of 442 articles. After an initial screening based on the reading of titles and abstracts, 161 articles were selected. These have been read in their entirety and their eligibility was evaluated based on the following inclusion criterion: the presence of at least one US characteristic of TPSUL within the article. A total of 31 articles met our criterion; an analysis of the references led to the identification of 7 additional articles, for a total of 38 works (Roy et al. 2004; Lai et al. 2018; Franco et al. 2023; Le et al. 2019; Chen et al. 2019; Marcotte‐Bloch et al. 2007; Gassama et al. 2017; Kim et al. 2013, 2022; Birchard et al. 2005; Shin and Kang 2021; Valentin 2009; Patil et al. 2015; Currie et al. 2013; Dhillon et al. 2023; Agrawal et al. 2023; Takeda and Nakamura 2016; Levai et al. 2019; Tsai et al. 2006; Anthimidis 2015; Yeh et al. 1999; Tawashi et al. 2023; Romano et al. 2021; Manchanda and Sharma 2021; Costa and Cunha 2020; Youssef 2018; Yamashita et al. 2022; Durai et al. 2022; Jadeja et al. 2017; Habib et al. 2016; Charles et al. 2017; Basso et al. 2017; Ardovino et al. 2011; Ward et al. 2011; Han et al. 2011; Joseph et al. 2023; Tavernaraki et al. 2020; Yasa et al. 2021). Most of the included articles are Case Reports (Roy et al. 2004; Le et al. 2019; Chen et al. 2019; Marcotte‐Bloch et al. 2007; Gassama et al. 2017; Kim et al. 2013, 2022; Shin and Kang 2021; Currie et al. 2013; Dhillon et al. 2023; Agrawal et al. 2023; Takeda and Nakamura 2016; Levai et al. 2019; Tsai et al. 2006; Anthimidis 2015; Yeh et al. 1999; Tawashi et al. 2023; Romano et al. 2021; Costa and Cunha 2020; Yamashita et al. 2022; Durai et al. 2022; Jadeja et al. 2017; Habib et al. 2016; Charles et al. 2017; Basso et al. 2017; Ardovino et al. 2011; Ward et al. 2011; Han et al. 2011; Joseph et al. 2023; Tavernaraki et al. 2020; Yasa et al. 2021); one work is a Case Series (Lai et al. 2018); the remaining articles discuss the US characteristics of TPSUL within broader contexts (Franco et al. 2023; Birchard et al. 2005; Valentin 2009; Patil et al. 2015; Youssef 2018). Given that the majority of included studies were case reports and small case series, a formal risk of bias assessment was not performed, as the aim of this review was primarily descriptive.

Of the 38 selected articles, 37 described US characteristics of a single clinical case of TPSUL (Roy et al. 2004; Franco et al. 2023; Le et al. 2019; Chen et al. 2019; Marcotte‐Bloch et al. 2007; Gassama et al. 2017; Kim et al. 2013, 2022; Birchard et al. 2005; Shin and Kang 2021; Valentin 2009; Patil et al. 2015; Currie et al. 2013; Dhillon et al. 2023; Agrawal et al. 2023; Takeda and Nakamura 2016; Levai et al. 2019; Tsai et al. 2006; Anthimidis 2015; Yeh et al. 1999; Tawashi et al. 2023; Romano et al. 2021; Manchanda and Sharma 2021; Costa and Cunha 2020; Youssef 2018; Yamashita et al. 2022; Durai et al. 2022; Jadeja et al. 2017; Habib et al. 2016; Charles et al. 2017; Basso et al. 2017; Ardovino et al. 2011; Ward et al. 2011; Han et al. 2011; Joseph et al. 2023; Tavernaraki et al. 2020; Yasa et al. 2021), while one article reported 5 cases (Lai et al. 2018). Consequently, a total of 42 cases were extrapolated. If certain US characteristics were not specified in the description, but at least one image was available in the article that allowed extrapolation of further data, three US experts—M.C.S., M.P., and A.P., who are also authors of this article—were asked to independently describe the lesion, without mutual influence. If the characteristics described were consistent among all three operators, they were included in the analysis.

The US characteristics were extrapolated, described, and standardized based on the consensus opinion of the MUSA (Morphological Uterus Sonographic Assessment) group (Van Den Bosch et al. 2015), while striving to faithfully represent what was stated by the various authors of the collected articles.

The process of identifying the studies is summarized using a flow diagram based on the “Preferred Reporting Items for Systematic Reviews and Meta‐Analyses” (PRISMA) model (Moher et al. 2009) as illustrated in Figure 5.

**FIGURE 5:**
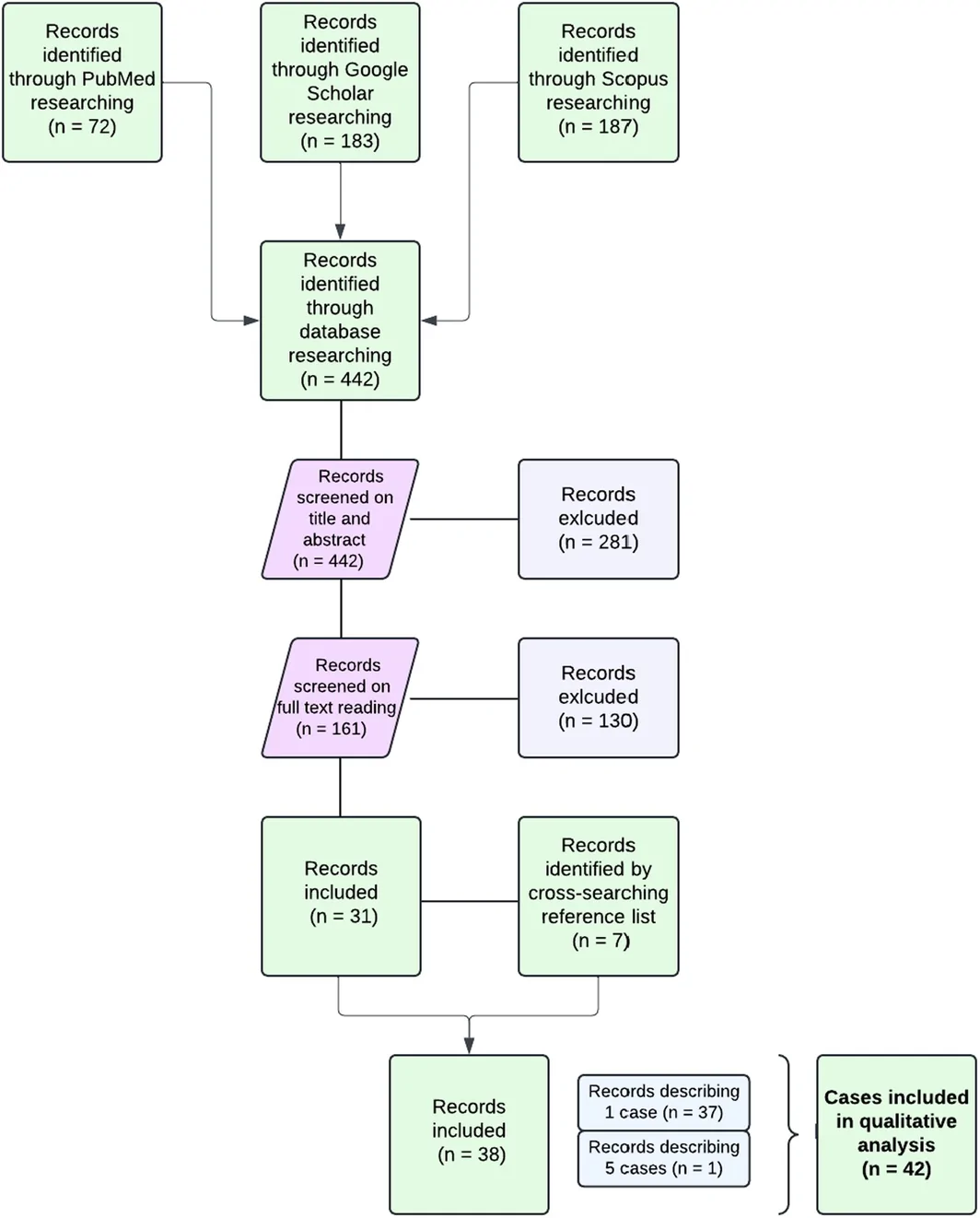
PRISMA flowchart illustrating the selection process of studies included in the systematic review. Records were identified through database searches (PubMed, Google Scholar, Scopus) and additional cross‐referencing. After screening titles, abstracts, and full texts, 38 studies reporting a total of 42 cases were included in the qualitative analysis.

## 4 Results

The reproductive phase of the patients in the studied population, the ultrasound techniques used, any additional imaging modalities employed for diagnosis, surgical intervention (if performed), and presence of a histopathological diagnosis are presented in Table 2.

**TABLE 2:** Overview of clinical profiles and diagnostic workup.

	*N*°	%
Reproductive status		
Childbearing age	35	83.3%
Menopause	3	7.15%
Premenarchal	1	2.4%
Pregnant status	14	33.3%
Unknown	3	7.15%
Modality of US scan		
Transvaginal	15	35.7%
Transabdominal	25	59.5%
Transrectal	2	4.8%
Unknown	4	9.5%
Other radiological methods		
TC	17	40.5%
MRI	15	35.7%
RX	1	2.4%
None	14	33.3%
Surgery		
Yes	40	95%
No	1	2.5%
Unknown	1	2.5%
Histopathological diagnosis		
Yes	30	71%
No	1	3%
Unknown	11	26%

*Note:* Reproductive phase of the patients in the studied population, ultrasound techniques used, additional imaging modalities employed for diagnosis, surgical intervention (if performed), and presence of a histopathological diagnosis.

Among the 42 cases, 35 (83.3%) were of childbearing age (Roy et al. 2004; Franco et al. 2023; Chen et al. 2019; Marcotte‐Bloch et al. 2007; Gassama et al. 2017; Kim et al. 2013, 2022; Birchard et al. 2005; Shin and Kang 2021; Patil et al. 2015; Currie et al. 2013; Dhillon et al. 2023; Agrawal et al. 2023; Takeda and Nakamura 2016; Levai et al. 2019; Tsai et al. 2006; Anthimidis 2015; Yeh et al. 1999; Tawashi et al. 2023; Romano et al. 2021; Costa and Cunha 2020; Youssef 2018; Durai et al. 2022; Jadeja et al. 2017; Habib et al. 2016; Charles et al. 2017; Basso et al. 2017; Ardovino et al. 2011; Han et al. 2011; Joseph et al. 2023; Yasa et al. 2021), including 14 pregnancies (33.3%) (Birchard et al. 2005; Shin and Kang 2021; Kim et al. 2022; Currie et al. 2013; Anthimidis 2015; Tawashi et al. 2023; Costa and Cunha 2020; Youssef 2018; Durai et al. 2022; Habib et al. 2016; Basso et al. 2017; Ardovino et al. 2011; Joseph et al. 2023; Yasa et al. 2021); 3 (7.15%) were postmenopausal (Le et al. 2019; Yamashita et al. 2022; Tavernaraki et al. 2020), 1 (2.4%) premenarchal (Manchanda and Sharma 2021), and 3 (7.15%) had unknown reproductive status (Lai et al. 2018; Ward et al. 2011).

The US examination was mainly transabdominal (25 cases, 59.5%) (Roy et al. 2004; Lai et al. 2018; Chen et al. 2019; Marcotte‐Bloch et al. 2007; Gassama et al. 2017; Shin and Kang 2021; Kim et al. 2022; Dhillon et al. 2023; Agrawal et al. 2023; Anthimidis 2015; Yeh et al. 1999; Tawashi et al. 2023; Manchanda and Sharma 2021; Costa and Cunha 2020; Durai et al. 2022; Habib et al. 2016; Charles et al. 2017; Basso et al. 2017; Ardovino et al. 2011; Joseph et al. 2023; Yasa et al. 2021), followed by transvaginal (15 cases, 35.7%) (Lai et al. 2018; Franco et al. 2023; Le et al. 2019; Chen et al. 2019; Marcotte‐Bloch et al. 2007; Kim et al. 2013; Patil et al. 2015; Dhillon et al. 2023; Takeda and Nakamura 2016; Anthimidis 2015; Romano et al. 2021; Yamashita et al. 2022; Durai et al. 2022; Jadeja et al. 2017; Ward et al. 2011) and transrectal (2 cases, 4.8%) (Romano et al. 2021; Han et al. 2011). In 4 cases (9.5%), the method was unknown (Birchard et al. 2005; Valentin 2009; Currie et al. 2013; Levai et al. 2019). Percentages exceed 100% as multiple techniques were sometimes used.

Besides US, additional imaging modalities were often used: CT in 17 cases (40,5%) (Roy et al. 2004; Lai et al. 2018; Franco et al. 2023; Le et al. 2019; Chen et al. 2019; Marcotte‐Bloch et al. 2007; Birchard et al. 2005; Shin and Kang 2021; Agrawal et al. 2023; Levai et al. 2019; Romano et al. 2021; Yamashita et al. 2022; Jadeja et al. 2017; Han et al. 2011; Tavernaraki et al. 2020), MRI in 15 (35.7%) (Franco et al. 2023; Marcotte‐Bloch et al. 2007; Kim et al. 2013, 2022; Birchard et al. 2005; Shin and Kang 2021; Patil et al. 2015; Dhillon et al. 2023; Takeda and Nakamura 2016; Tawashi et al. 2023; Manchanda and Sharma 2021; Costa and Cunha 2020; Yamashita et al. 2022; Durai et al. 2022; Joseph et al. 2023), and X‐ray in 1 (2.4%) (Han et al. 2011). In 14 cases (33.3%) (Lai et al. 2018; Gassama et al. 2017; Valentin 2009; Currie et al. 2013; Tsai et al. 2006; Anthimidis 2015; Yeh et al. 1999; Habib et al. 2016; Charles et al. 2017; Basso et al. 2017; Ardovino et al. 2011; Ward et al. 2011; Yasa et al. 2021), only US was performed. Percentages exceed 100% as multiple methods were sometimes employed.

Surgery was performed in 40 of 42 cases (95%) (Roy et al. 2004; Lai et al. 2018; Franco et al. 2023; Le et al. 2019; Chen et al. 2019; Marcotte‐Bloch et al. 2007; Gassama et al. 2017; Kim et al. 2013, 2022; Birchard et al. 2005; Shin and Kang 2021; Valentin 2009; Currie et al. 2013; Dhillon et al. 2023; Agrawal et al. 2023; Takeda and Nakamura 2016; Levai et al. 2019; Tsai et al. 2006; Anthimidis 2015; Yeh et al. 1999; Tawashi et al. 2023; Romano et al. 2021; Manchanda and Sharma 2021; Youssef 2018; Yamashita et al. 2022; Durai et al. 2022; Jadeja et al. 2017; Habib et al. 2016; Charles et al. 2017; Basso et al. 2017; Ardovino et al. 2011; Ward et al. 2011; Han et al. 2011; Joseph et al. 2023; Tavernaraki et al. 2020; Yasa et al. 2021), while one case was unspecified (2.5%) (Patil et al. 2015), and one was managed conservatively (2.5%) (Costa and Cunha 2020). A histopathological diagnosis was reported in 30 cases (71%) (Roy et al. 2004; Lai et al. 2018; Le et al. 2019; Chen et al. 2019; Marcotte‐Bloch et al. 2007; Kim et al. 2013, 2022; Shin and Kang 2021; Valentin 2009; Dhillon et al. 2023; Agrawal et al. 2023; Levai et al. 2019; Tsai et al. 2006; Tawashi et al. 2023; Romano et al. 2021; Manchanda and Sharma 2021; Yamashita et al. 2022; Durai et al. 2022; Jadeja et al. 2017; Habib et al. 2016; Charles et al. 2017; Basso et al. 2017; Ardovino et al. 2011; Ward et al. 2011; Han et al. 2011; Joseph et al. 2023).

Table 3 presents the US characteristics of all cases found in the literature. The term “Described” refers to cases where the characteristics were reported by the authors or unanimously identified by the three US technicians mentioned above. The term “Unknown” indicates cases where a specific US characteristic was neither described by the authors nor inferable from the article's images.

**TABLE 3:** Summary of ultrasound features.

US feature		Findings	%	Description	Data
Position		Described	34 (77%)	Right	7 (20.5%)
				Left	7 (20.5%)
				Fundic	7 (20.5%)
				Anterior	6 (18%)
				Posterior	7 (20.5%)
		Unknown	10 (23%)		
Largest diameter (mm)		Described	36 (86%)	Average	105.31
				Median	103.00
		Unknown	6 (14%)		
Enlargement		Described	8 (19%)	Enlarged	8 (100%)
		Unknown	34 (81%)		
Free fluid		Described	28 (67%)	Present	16 (57%)
				Absent	12 (43%)
		Unknown	14 (33%)		
Probe tenderness		Described	12 (29%)	Present	12 (100%)
				Absent	0 (0%)
		Unknown	30 (71%)		
Myoma features	Echostructure	Described	28 (67%)	Heterogeneous	25 (81%)
				Homogeneous	1 (3%)
				Hypoechoic	5 (16%)
		Unknown	14 (33%)		
	Margins	Described	22 (52%)	Regular	20 (90%)
				Irregular	2 (10%)
		Unknown	20 (48%)		
	Vascularity	Described	19 (45%)	CS 1	13 (68%)
				CS 2	6 (32%)
				CS 3	0 (0%)
				CS 4	0 (0%)
		Unknown	23 (56%)		
	Acoustic shadows	Described	16 (38%)	Present	15 (94%)
				Absent	1 (6%)
		Unknown	26 (62%)		
	Cystic areas	Described	20 (48%)	Present	11 (55%)
				Absent	9 (45%)
		Unknown	22 (52%)		
	Cystic walls	Described	9 (21%)	Regular	0 (0%)
				Irregular	9 (100%)
		Unknown	33 (79%)		
	Cyst content	Described	9 (21%)	Anechoic	4 (44%)
				Low‐level	2 (22%)
				Ground‐glass	1 (11%)
				Mixed	2 (22%)
		Unknown	33 (79%)		
	Pedicle	Described	29 (69%)	Seen	8 (28%)
				Unseen	21 (72%)
				Torsion signs	1 (12.5%)
				Cessation of vascular flow at the proximal portion	2 (25%)
		Unknown	13 (31%)		
	Calcifications	Described	17 (40%)	Present	4 (24%)
				Absent	13 (76%)
		Unknown	25 (60%)		

*Note:* Summary of the reproductive phase of the patients, type of ultrasound performed, additional radiological investigations, surgical treatment (if any), and histopathological confirmation across all cases included in the systematic review.

*Position*. The leiomyoma's position in relation to the uterus was reported in 34 of 42 cases (77%): 7 were right‐sided (20.5%) (Le et al. 2019; Kim et al. 2022; Agrawal et al. 2023; Tawashi et al. 2023; Basso et al. 2017; Joseph et al. 2023; Yasa et al. 2021), 7 left‐sided (20.5%) (Tsai et al. 2006; Romano et al. 2021; Manchanda and Sharma 2021; Durai et al. 2022; Jadeja et al. 2017; Habib et al. 2016; Basso et al. 2017), 7 fundal (20.5%) (Gassama et al. 2017; Dhillon et al. 2023; Anthimidis 2015; Youssef 2018; Charles et al. 2017; Ardovino et al. 2011; Ward et al. 2011), 6 anterior (18%) (Roy et al. 2004; Marcotte‐Bloch et al. 2007; Shin and Kang 2021; Currie et al. 2013; Basso et al. 2017; Tavernaraki et al. 2020), and 7 posterior (20.5%) (Chen et al. 2019; Kim et al. 2013; Patil et al. 2015; Takeda and Nakamura 2016; Levai et al. 2019; Yamashita et al. 2022; Han et al. 2011) (Table 3).

*Size*. The largest diameter of the TPSUL ranged from 55 to 200 mm; the average was 105.31 mm, while the median was 103 mm.

*Enlargement*. Fibroid volume change was reported in 8 out of 42 cases (19%) (Le et al. 2019; Currie et al. 2013; Levai et al. 2019; Yeh et al. 1999; Habib et al. 2016; Basso et al. 2017; Ward et al. 2011; Tavernaraki et al. 2020), with 100% showing enlargement. In 2 out of 42 cases (4.8%) (Levai et al. 2019; Habib et al. 2016), the volume increase was unquestionably attributable to torsion.

*Free fluid*. The presence of pelvic free fluid was described in 28 out of 42 cases (67%): in 16 cases (57%) it was present (Roy et al. 2004; Franco et al. 2023; Le et al. 2019; Chen et al. 2019; Kim et al. 2013; Patil et al. 2015; Currie et al. 2013; Dhillon et al. 2023; Levai et al. 2019; Anthimidis 2015; Yeh et al. 1999; Romano et al. 2021; Durai et al. 2022; Han et al. 2011; Tavernaraki et al. 2020; Yasa et al. 2021) and in 12 cases (43%) it was absent (Marcotte‐Bloch et al. 2007; Shin and Kang 2021; Kim et al. 2022; Agrawal et al. 2023; Takeda and Nakamura 2016; Tawashi et al. 2023; Manchanda and Sharma 2021; Costa and Cunha 2020; Yamashita et al. 2022; Jadeja et al. 2017; Charles et al. 2017; Ardovino et al. 2011). Two studies (7.1%) described massive hemoperitoneum (Levai et al. 2019; Jadeja et al. 2017).

*Probe tenderness*. Tenderness upon targeted probe pressure was reported in 12 of 48 cases (29%) (Gassama et al. 2017; Kim et al. 2022; Patil et al. 2015; Agrawal et al. 2023; Levai et al. 2019; Tsai et al. 2006; Yeh et al. 1999; Romano et al. 2021; Youssef 2018; Habib et al. 2016; Ward et al. 2011; Yasa et al. 2021). No cases explicitly stated its absence.

*Echostructure*. The echostructure of TPSUL was reported in 28 out of 42 articles (67%): in 1 case (3%) the fibroid was described as “homogeneous” (Tsai et al. 2006), in 25 cases (81%) as “heterogeneous” (Roy et al. 2004; Franco et al. 2023; Le et al. 2019; Chen et al. 2019; Marcotte‐Bloch et al. 2007; Kim et al. 2013, 2022; Shin and Kang 2021; Valentin 2009; Dhillon et al. 2023; Agrawal et al. 2023; Takeda and Nakamura 2016; Levai et al. 2019; Yeh et al. 1999; Romano et al. 2021; Manchanda and Sharma 2021; Costa and Cunha 2020; Youssef 2018; Yamashita et al. 2022; Jadeja et al. 2017; Charles et al. 2017; Basso et al. 2017; Han et al. 2011; Joseph et al. 2023; Yasa et al. 2021), and in 5 cases (16%) as “hypoechoic” (Patil et al. 2015; Dhillon et al. 2023; Levai et al. 2019; Jadeja et al. 2017; Habib et al. 2016). In 3 cases the fibroid was described as both “hypoechoic” and “heterogeneous” (Dhillon et al. 2023; Levai et al. 2019; Jadeja et al. 2017); as a result, the total frequency of these characteristics exceeds 28, reaching 31.

*Margins*. The margins of TPSUL were evaluated in 22 out of 42 cases (52%). In 20 cases (90%) the margins were classified as regular (Roy et al. 2004; Franco et al. 2023; Chen et al. 2019; Kim et al. 2013, 2022; Shin and Kang 2021; Valentin 2009; Patil et al. 2015; Dhillon et al. 2023; Takeda and Nakamura 2016; Levai et al. 2019; Tsai et al. 2006; Yeh et al. 1999; Tawashi et al. 2023; Romano et al. 2021; Manchanda and Sharma 2021; Jadeja et al. 2017; Charles et al. 2017; Han et al. 2011; Joseph et al. 2023), while in 2 cases (10%) they were irregular (Agrawal et al. 2023; Yamashita et al. 2022).

*Vascularization*. Color and Power Doppler evaluations were classified according to the Color Score (CS) described by the MUSA group consensus (Van Den Bosch et al. 2015). Vascularization was assessed in 19 out of 42 cases (45%). Among these, 13 cases (68%) were reported as absent/avascular (CS 1) (Roy et al. 2004; Franco et al. 2023; Le et al. 2019; Marcotte‐Bloch et al. 2007; Birchard et al. 2005; Valentin 2009; Kim et al. 2022; Patil et al. 2015; Dhillon et al. 2023; Takeda and Nakamura 2016; Youssef 2018; Joseph et al. 2023; Yasa et al. 2021), while 6 cases (32%) showed minimal vascularization (CS 2) (Agrawal et al. 2023; Levai et al. 2019; Anthimidis 2015; Romano et al. 2021; Manchanda and Sharma 2021; Durai et al. 2022). No cases exhibited moderate (CS 3) or marked vascularization (CS 4). In 3 out of 19 cases (16%) where vascularization was present, a CS downscaling following torsion was reported (Savelli and Cristina Scifo 2015; Testa et al. 2016; Van Den Bosch et al. 2015).

*Acoustic shadows*. Acoustic shadows were assessed in 16 cases (38%); in 1 case (2.4%) they were absent (Valentin 2009), while in 15 cases they were present (94%). To be precise, through‐lesion or “fan‐shaped” acoustic shadows were identified in 8 articles (Roy et al. 2004; Le et al. 2019; Dhillon et al. 2023; Yeh et al. 1999; Romano et al. 2021; Charles et al. 2017; Ward et al. 2011; Han et al. 2011), while posterior acoustic shadows were reported in 14 articles (Le et al. 2019; Shin and Kang 2021; Dhillon et al. 2023; Agrawal et al. 2023; Takeda and Nakamura 2016; Tsai et al. 2006; Yeh et al. 1999; Romano et al. 2021; Yamashita et al. 2022; Jadeja et al. 2017; Charles et al. 2017; Ward et al. 2011; Han et al. 2011; Joseph et al. 2023).

*Cystic areas*. The presence of cystic areas was analyzed in 20 out of 42 cases (48%). They were present in 11 cases (55%) (Franco et al. 2023; Chen et al. 2019; Shin and Kang 2021; Valentin 2009; Kim et al. 2022; Agrawal et al. 2023; Levai et al. 2019; Romano et al. 2021; Jadeja et al. 2017; Charles et al. 2017; Basso et al. 2017) and absent in 9 cases (45%) (Roy et al. 2004; Le et al. 2019; Kim et al. 2013; Patil et al. 2015; Dhillon et al. 2023; Takeda and Nakamura 2016; Anthimidis 2015; Yeh et al. 1999; Manchanda and Sharma 2021). Among the 11 cases, the cystic wall was described in 9 (82%) and was irregular in all cases (100%) (Chen et al. 2019; Shin and Kang 2021; Valentin 2009; Kim et al. 2022; Agrawal et al. 2023; Levai et al. 2019; Romano et al. 2021; Jadeja et al. 2017; Charles et al. 2017). The echogenicity of the cystic content was reported in 9 cases (82%): it was anechoic in 4 cases (44%) (Valentin 2009; Agrawal et al. 2023; Levai et al. 2019; Jadeja et al. 2017), “low‐level” in 2 cases (22%) (Kim et al. 2022; Charles et al. 2017), “ground‐glass” in 1 case (11%) (Chen et al. 2019), and “mixed” in 2 cases (22%) (Shin and Kang 2021; Romano et al. 2021).

*Vascular pedicle*. The vascular pedicle was evaluated in 29 out of 42 cases (69%). Among these, it was visualized in 8 cases (28%) (Le et al. 2019; Dhillon et al. 2023; Agrawal et al. 2023; Takeda and Nakamura 2016; Levai et al. 2019; Tawashi et al. 2023; Manchanda and Sharma 2021; Charles et al. 2017), while in 21 cases (72%), it was not visualized (Roy et al. 2004; Chen et al. 2019; Marcotte‐Bloch et al. 2007; Gassama et al. 2017; Kim et al. 2013, 2022; Birchard et al. 2005; Shin and Kang 2021; Patil et al. 2015; Currie et al. 2013; Tsai et al. 2006; Yeh et al. 1999; Romano et al. 2021; Durai et al. 2022; Jadeja et al. 2017; Habib et al. 2016; Ward et al. 2011; Han et al. 2011; Joseph et al. 2023; Tavernaraki et al. 2020; Yasa et al. 2021). In one article, the pedicle appeared twisted (12.5%) (Tawashi et al. 2023), while in two articles, blood flow seemed to stop at the proximal portion of the pedicle using Doppler (25%) (Le et al. 2019; Takeda and Nakamura 2016).

*Calcifications*. The presence or absence of calcifications was determined in 17 out of 42 cases (40%). Among these, calcifications were present in 4 cases (24%) (Romano et al. 2021; Yamashita et al. 2022; Jadeja et al. 2017; Charles et al. 2017), while in 13 cases (76%), they were absent (Roy et al. 2004; Le et al. 2019; Chen et al. 2019; Kim et al. 2013; Valentin 2009; Patil et al. 2015; Dhillon et al. 2023; Agrawal et al. 2023; Takeda and Nakamura 2016; Levai et al. 2019; Anthimidis 2015; Yeh et al. 1999; Manchanda and Sharma 2021).

## 5 Discussion

In 83.3% of the articles, the patients were of reproductive age, 40% of whom were pregnant. This finding corroborates the well‐established fact that fibroid growth is influenced by estrogenic and progestogenic stimulation, making degenerative changes and complications less likely in premenarchal or postmenopausal patients. In addition, several studies suggest that fibroids tend to grow during pregnancy (Ye et al. 2023; Vitagliano et al. 2018). In our case report, the patient was 21 weeks pregnant. No prior ultrasound images were available to confirm whether the fibroid had grown during pregnancy.

In 95% of cases, patients underwent surgical treatment. Surgical intervention is often required in cases of TPSUL, as these frequently constitute a surgical emergency (Gupta and Manyonda 2009; Roy et al. 2004; Testa et al. 2016; Franco et al. 2023; Le et al. 2019). However, in certain circumstances, such as during pregnancy, conservative management may be considered (Joseph et al. 2023). In our case report, the patient was 21 weeks pregnant, and conservative treatment allowed for the normal progression of the pregnancy.

We discuss below the ultrasound characteristics of TPSUL found in the literature that we consider most useful for making an ultrasound diagnosis, comparing them with those observed in our case report.

*Position*. Nearly identical percentages have been reported for all locations relative to the uterus: it can be concluded that the position of fibroids does not influence the likelihood of torsion. In our case report, the TPSUL was located on the right lateral uterine wall.

*Size*. The average largest diameter was 105.31 mm. Although no specific cutoff is established to define a fibroid as “large,” it can be inferred that larger pedunculated subserosal fibroids are more prone to torsion. Additionally, larger fibroids are less likely to spontaneously resolve torsion, unlike smaller ones (Tsai et al. 2006). In the case report, the largest diameter of the TPSUL was 80 mm.

*Enlargement*. In all reported cases, leiomyomas exhibited volumetric enlargement compared to prior evaluations. In two studies (Levai et al. 2019; Habib et al. 2016), the volume increase followed torsion, likely resulting from venous stasis and inflammation (Testa et al. 2016). Four studies (Le et al. 2019; Currie et al. 2013; Basso et al. 2017; Ward et al. 2011) also reported fibroid enlargement, though it was unclear whether it preceded or followed torsion. In these instances, the enlargement may have predisposed the fibroid to torsion, although inflammation secondary to torsion could also have contributed to the increase in size. Regardless of the underlying cause, an increase in fibroid size may support the diagnosis of torsion. In our case report, determining whether the fibroid's largest diameter increased before or after torsion was not possible due to the lack of prior imaging.

*Free fluid*. The presence of free fluid was seen in a significant percentage of cases (57%). Subserosal fibroids are known to cause small amounts of ascites due to peritoneal irritation (Jimerson 1973). Additionally, the inflammatory processes associated with torsion can contribute to the formation of pelvic fluid (Yaguchi et al. 2020), although it is a nonspecific finding occurring in various clinical conditions. In our case report, a minimal amount of pelvic free fluid was detected.

*Probe tenderness*. Tenderness upon targeted US probe pressure was reported in all cases where it was assessed. This is a nonspecific clinical sign that may suggest an acute condition. In our case report, tenderness was elicited by US probe pressure.

*Echostructure*. A heterogeneous echostructure was observed in 81% of TPSUL cases, reflecting histopathological findings such as haemorrhagic degeneration and tissue edema. A markedly heterogeneous echostructure was also seen in our case report.

*Margins*. Margins were classified as regular in 90% of cases in the literature, as in our case report.

*Vascularization*. Torsion reduces blood supply to the fibroid, leading to ischemia, which is reflected in Doppler flowmetry findings as absent or diminished vascularization compared to prior observations (Savelli and Cristina Scifo 2015; Testa et al. 2016; Van Den Bosch et al. 2015). Referring to the CS described in the consensus opinion of the MUSA group (Van Den Bosch et al. 2015), TPSUL were reported as avascular (CS 1) in 68% of cases and with poor vascularization (CS 2) in the remaining 32%. In our case report, the fibroid's vascularization was absent (CS 1) (Figures 2 and 4).

*Acoustic shadows*. Acoustic shadows, either posterior or through‐lesion, were identified in 94% of assessed cases. In our case report, thin acoustic shadows were observed, both posterior and through‐lesion. Notably, the through‐lesion shadows appeared slightly irregular compared to the classic “fan‐shaped” shadows of uncomplicated leiomyomas, likely due to the markedly heterogeneous echostructure of the TPSUL (Figure 3).

*Cystic areas*. Cystic areas are signs of degeneration due to ischemia. They were reported in approximately 55% of cases: their presence may suggest the diagnosis of TPSUL, albeit with limited sensitivity. Their absence in some cases may be explained by an early stage of acute torsion, where degenerative changes have not yet developed (Testa et al. 2016). When present, cystic areas most often exhibited irregular borders and hypoechoic or anechoic content. In our case report, cystic areas were identified, showing irregular borders and low‐level content (Figures 1 and 3).

*Vascular pedicle*. The fibroid pedicle was visible in 28% of cases. In two cases, blood flow appeared interrupted in the proximal portion (Le et al. 2019; Takeda and Nakamura 2016); in another, the venous flow was absent at the pedicle level, while arterial flow was detected on spectral Doppler (Dhillon et al. 2023). One article described the pedicle as twisted (Tawashi et al. 2023). Although direct signs of torsion are difficult to detect, Doppler findings can be highly suggestive. Thus, assessing the fibroid pedicle is always advisable. In our case, the vascular pedicle was visible on Doppler ultrasound and appeared twisted, resembling the “Whirlpool sign,” a term commonly used to describe the ultrasound appearance of torsion in adnexal structures (Adu‐Bredu et al. 2021). In this context, it was used by an experienced operator as a differential diagnostic clue to suggest torsion of the myoma. For this reason, we considered it relevant to report and describe this finding, as it may represent a potentially useful sign for clinicians in similar scenarios.

*Calcifications*. Calcifications were described in only four articles. This is not surprising, as calcification represents the final stage of fibroid degeneration following torsion (Nicholson et al. 2001). Acute pelvic pain, a common symptom of TPSUL, typically prompts early US evaluation before calcification processes have developed. Therefore, calcifications are not among the typical US characteristics of TPSUL. In our case report, no calcifications were visible.

### 5.1 Key Ultrasound Features and Diagnostic Implications

Overall, no single ultrasound feature appears to be pathognomonic of TPSUL. Rather, the diagnosis should be suspected based on a combination of findings, particularly heterogeneous echotexture, absent or reduced vascularization, presence of cystic areas, and, when visible, abnormalities of the vascular pedicle (e.g., “whirlpool sign”). These features, although individually nonspecific, may become clinically relevant when associated with acute abdominal pain and a known or suspected pedunculated subserosal fibroid. In this context, ultrasound should be interpreted as a dynamic and integrative tool, supporting clinical suspicion and guiding timely management rather than providing a definitive diagnosis. To facilitate practical recognition, these features are summarized in an iconographic overview (Figure 6).

**FIGURE 6:**
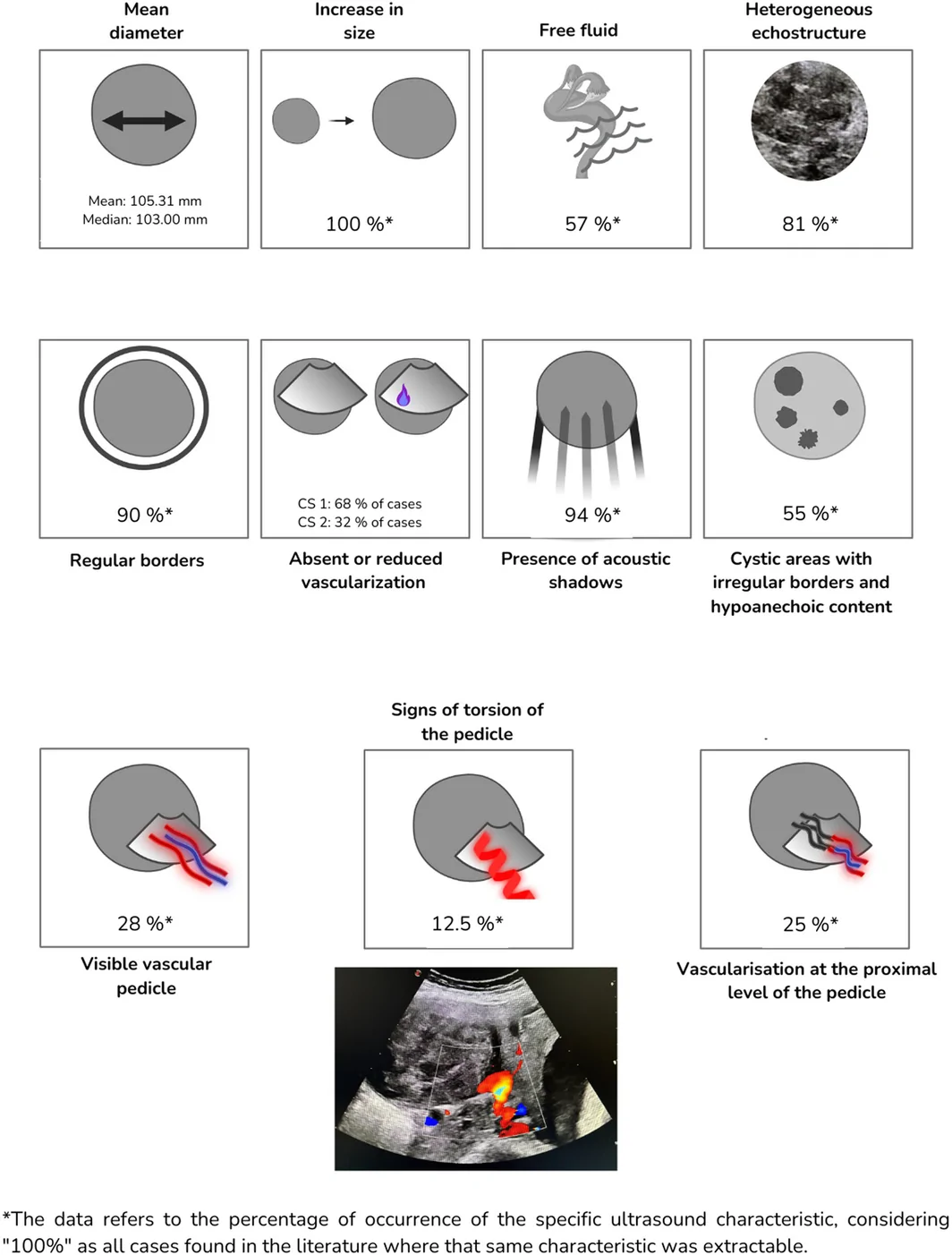
Iconographic summary of the study conclusions. Graphical representation of the main ultrasound features observed in cases of twisted pedunculated subserosal uterine leiomyomas (TPSUL). Each icon illustrates a specific sonographic sign along with the corresponding frequency of occurrence reported in the literature. These findings support the potential diagnostic value of ultrasound in identifying TPSUL preoperatively.

### 5.2 Strengths and limitations

This study represents, to our knowledge, the first systematic review specifically focused on the ultrasound characteristics of TPSUL, including 42 cases overall. It provides a structured synthesis of a rare but clinically relevant condition that may present insidiously and lead to acute abdomen. The integration of an illustrative case, ultrasound images, and an iconographic summary further enhances the practical applicability of the findings, while the use of the MUSA consensus allows standardized description of ultrasound features. A key limitation of this review is the high proportion of missing or unreported variables across the included studies, reflecting the heterogeneous and often incomplete nature of case report‐based literature. In addition, no formal assessment of inter‐observer agreement among expert reviewers was performed. Moreover, the available evidence consists almost exclusively of case reports, with only one case series, making this review unconventional compared with traditional systematic reviews and limiting the possibility of quantitative analysis, formal risk of bias assessment, and definition of diagnostic criteria. Therefore, the conclusions remain mainly qualitative and should be interpreted with caution.

## 6 Conclusions

TPSUL represent a rare but clinically relevant condition that should be considered in the differential diagnosis of acute abdomen, particularly in pregnant patients or in those with a known history of uterine fibroids. According to our findings, the position of the fibroid does not appear to influence the risk of torsion, whereas larger size may represent a predisposing factor. On ultrasound, TPSUL typically present as enlarged fibroids with heterogeneous echotexture, regular margins, posterior or fan‐shaped acoustic shadowing, and internal cystic areas with irregular walls and hypoechoic or anechoic content. The identification of a vascular pedicle, especially when showing features suggestive of torsion such as the “whirlpool sign,” may further support the diagnosis.

Despite these findings, no single ultrasound feature is diagnostic, and the condition remains challenging to identify preoperatively. Ultrasound should therefore be interpreted in conjunction with clinical presentation to support timely diagnosis and management. This work provides a foundation for improving recognition of TPSUL and for future efforts toward diagnostic standardization.

## Author Contributions

M.P. and A.P. were responsible for the selection of the articles included in the systematic literature review, recruited the patient case report, and extracted the data. P.P. coordinated the work and co‐wrote the manuscript with M.P. E.G., M.C.S., and G.C. conceived the study and contributed to the revision and proofreading of the manuscript throughout the writing process.

## Funding

The authors have nothing to report.

## Disclosure

This systematic review is based exclusively on data already available in the scientific literature and retrieved from the databases specified in the Methods section. All information reported in the manuscript is supported by clearly identified references. Therefore, no additional permission to reproduce material from other sources was required.

## Ethics Statement

The Ethical Committee of the Emilia‐Center Area (CE‐AVEC) reviewed the Clinical Study Submission form “Case Report” and the Informed Consent during the online meeting on April 18, 2024.

## Consent

Written informed consent was obtained from the patient for publication of the details of their medical case and any accompanying images. The patient of the Case Report signed an Informed Consent on April 2, 2024, at Ospedale Maggiore di Bologna, Italy.

## Conflicts of Interest

The authors declare no conflicts of interest.

## Data Availability

The data that support the findings of this study are available on request from the corresponding author. The data are not publicly available due to privacy or ethical restrictions.
